# Scientific conferences, socialization, and the Covid-19 pandemic: A conceptual and empirical enquiry

**DOI:** 10.1177/03063127221138521

**Published:** 2023-01-12

**Authors:** Harry Collins, Willow Leonard-Clarke, Will Mason-Wilkes

**Affiliations:** 1 School of Social Sciences, Cardiff University, Cardiff, UK; 2 Institute for STEMM in Culture and Society, University of Birmingham, Birmingham, UK

**Keywords:** scientific conferences, Covid-19 pandemic, face-to-face communication in science, remote conferences, algorithmical model, enculturational model, science and democracy

## Abstract

Since the 1970s social analysts have seen communication between scientists not solely as information exchange (the algorithmical model), but as a process of socialization into overlapping and mutually embedded scientific domains (the enculturational model). Under the algorithmical model, the impact of the Covid-19 shutdown on travel would be easily remedied by replacing face-to-face communication with online platforms. Conferences and similar gatherings are costly, elitist, and environmentally damaging, but under the enculturational model abandoning them could be disastrous for science, which depends on the development of cross-national trust and mutual agreements through face-to-face interaction and, in turn, disastrous for science’s role in democracy. We explore the problem theoretically and empirically, arguing against recent proposals from some scientists for the wholesale and permanent replacement of conferences with remote communication.

Scientific communication at conferences and other meetings has a new salience with the Covid-related lockdown of face-to-face (F2F) gatherings and their replacement with remote communication (R2R) over the internet.^1^ Here we analyse this change and report on a study of the first 6 months of experience of Covid-constrained socialization among two groups of physicists studying gravitational waves and photonics. We set this data in the context of previous work by one of the authors on tacit knowledge transfer in gravitational wave physics. We have taken advantage of the opportunity provided by the Covid-19 pandemic to observe a brief but major social change in how science is conducted, a change that some scientists hope will become permanent.

Even before 2020, some scientists extolled the advantages of remote conferences, which include speedier and more regular interaction with remote colleagues; a widening and levelling out of the scientific community as new groups and individuals are drawn in who are unable to travel long distances because of the expense, visa requirements, disability/health, or because they are local caregivers; and a radical reduction in science’s carbon footprint. Some scientists have issued calls for or predictions of the permanent abolition of the conference circuit following the end of pandemic restrictions (e.g. Sarabipour et al., 2021; Woodgett, 2020).^2^ Climate change considerations in particular have led some members of the academic community to foreswear any airplane travel even when this could damage their careers.^3^

STS scholars have long argued that communication among natural scientists serves the purpose of socializing new scientists into domains characterized by bodies of tacit knowledge and taken-for-granted procedures, and that assumptions are transferred and maintained through personal interaction. This *enculturational* model of scientific communication stands in contrast to the *algorithmical* model, which sees scientific communication as simply a means of information exchange. If scientific communication was solely about information exchange, a switch from F2F to R2R would be largely beneficial. If, on the other hand, the crucial feature of scientific communication is socialization and developing trust relationships, the suspension of F2F scientific meetings, if made permanent, would have damaging consequences not only for individuals but for the very nature of science. We will argue that, while long-term changes to address climate and inegalitarianism^4^ concerns are appropriate, they must take into account the more subtle sociological role of F2F meetings if science is to retain its crucial character. Safeguarding sound science is a social responsibility just as much as safeguarding the climate, indeed the latter is parasitic on the former. A suitable compromise, compatible with the principles put forward in this paper, would be to limit long-haul travel to scientific meetings to those which are organized to enhance egalitarianism and provide significant opportunities for personal interaction.

## Adventurous science

We concentrate here on the effects on the kind of science which aims to challenge existing knowledge or advance knowledge in radical and often contested ways. We will call this *adventurous science*. Kuhn’s (1962) Structure of Scientific Revolutions introduced the distinction between *normal* and *revolutionary science*, but *adventurous* science includes the more exploratory aspect of normal science. Thus, gravitational wave detection was never a revolutionary science since it rested upon Einstein’s theories of relativity and its experiments were based on known principles, but the waves were so weak that a large proportion of the physics community vigorously resisted the funding of Earth-based detectors and remained sceptical about their potential for around half a century – this is adventurous science. Our own work in progress using molecular biology as an example argues that routine science can be much more readily described using an information exchange model which is less dependent on the building of new trusting communities. Indeed, here we suggest there is some difference in this respect between our two exemplary fields of gravitational wave detection and photonics, with photonics being slightly more routine. In work in press, we suggest that many domains of molecular biology are even more routine, exhibiting what we will call *hypernormality*, which is a form of science that works well with information exchange alone, with little or no need for culture building. This means such domains can manage adequately with remote communication because their methods and assumptions have been widely accepted (sometimes to the point of automation) and new entrants can learn the practices and taken-for-granted understandings of the field through graduate training (H. Collins, Shrager, et al., 2022 forthcoming).^5^

## Science, truth, and trust

**Figure 1.**
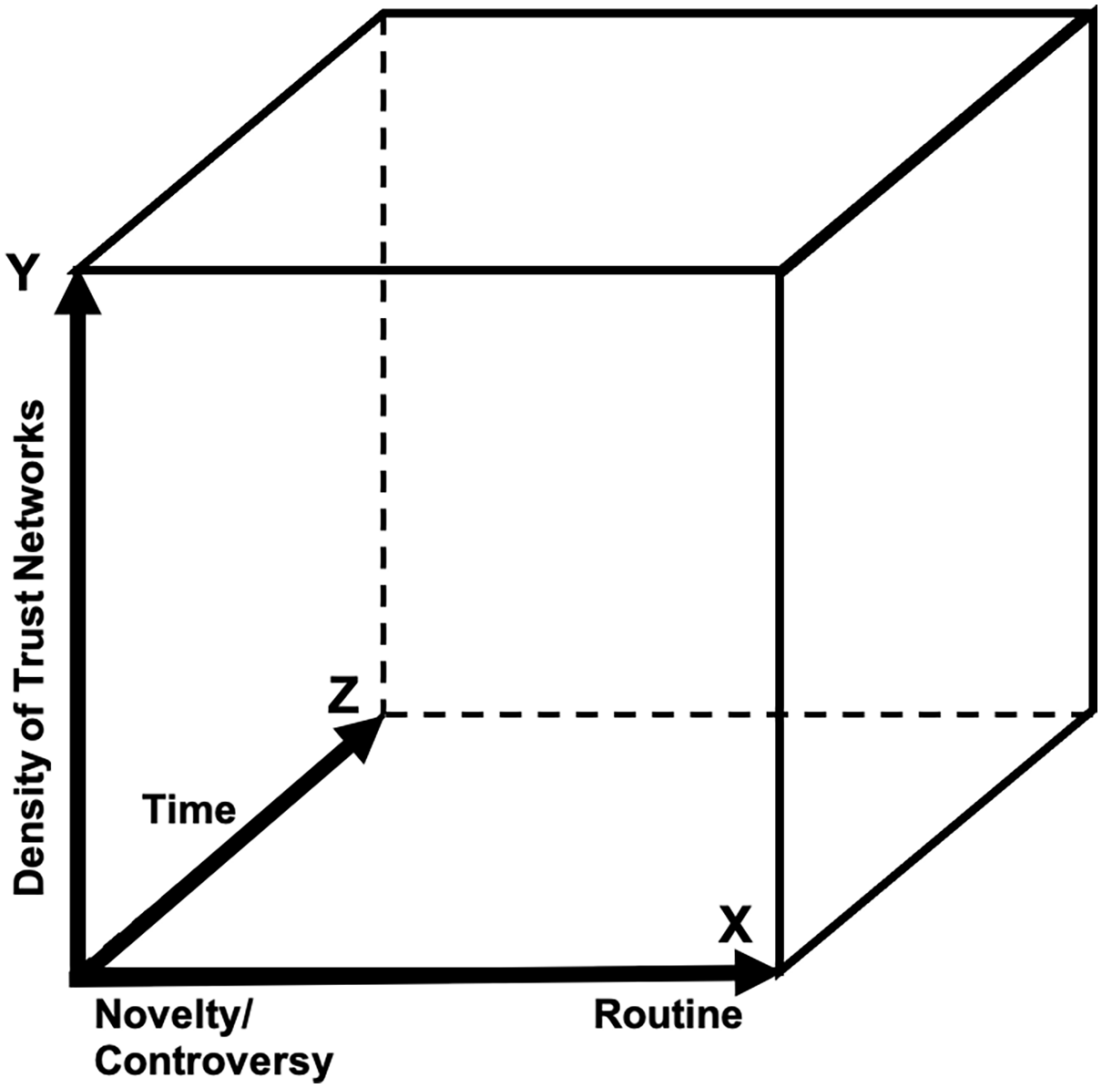
Replacement of F2F with R2R over time.

Looked at in the widest perspective, a defining characteristic of the institution of science – its principal formative aspiration – is to generate truth.^6^ This truth is intended to be universally accepted, at least by those who are competent to understand and test it.^7^ In adventurous sciences, the necessary competences are likely to be found only in narrow groups of researchers working in esoteric domains with their own esoteric *domain languages* and though the groups may be bigger in less adventurous sciences, the body of competent testers is still small compared to the general population (e.g. H. Collins, 2011). Adventurous science, therefore, builds small expert groups with shared cultures and carefully controlled boundaries, whose membership is recruited, at least in principle, irrespective of regular cultural or national identities because the aim is to generate universally acceptable truths.^8^ Among other things, in the adventurous sciences, scientific conferences and other such interpersonal meetings enable common, if narrow, scientific socialization into these esoteric domains. This is the most important characteristic of science that could be lost post-pandemic: in so far as science acts as a role model for, and a check and balance on, decision-making under uncertainty in democracies, this would have damaging consequences for society as a whole: the most culturally prestigious model for decision making in pluralist democracies – science – could start to take on the characteristics of social media with popularity replacing carefully nurtured trust.^9^ These points are summarized in diagrammatic form in Figure 1.

The front plane of the three-dimensional space represented in Figure 1 represents the moment of lockdown, when F2F ceases and is replaced by R2R, while the effects unfold over time as represented by the Z-axis going into the page. In this paper we use the term ‘lockdown’ to represent the shutting down of F2F interaction between scientific researchers. The X-axis is the extent to which a science is characterized by adventurous science (i.e. radically novel, beset by controversy of the sort that is best resolved by F2F debate, and/or requiring consensus building coextensive with the growth of new trusting relationships), as opposed to settled and routine science, which is more likely to work with an information exchange model, at least in the short term. The Y-axis represents the extent to which the domain is initially characterized by dense taken-for-granted and trusting relationships: other things being equal, the greater the density of trust relations at the outset the less will the negative impact of a shift to R2R be felt in the short term.^10^

The issue is difficult to investigate because, in the short term, a switch from F2F to R2R may be experienced as little more than an inconvenience. Most scientists in a domain will already have completed apprenticeships and developed dense, trusting, international networks along with established cultural understandings (though, as we will see, the impact on new apprentices appears to be immediate and widely noticed). Therefore, other things being equal, the significant impact of a shutdown of opportunities to meet personally is likely to make itself felt only gradually on science as a whole and the most serious effects may not be felt until a new generation has grown up without interpersonal networks. Effects that require a generation or more to show themselves cannot be empirically investigated for now, only theorized, hence, the balance of theory and empirical results in this paper.^11^ We need to theorize and start to investigate the problem now, however, because to leave it until proof of a more complete empirical kind is available could leave us trying the bolt the stable door after the horse has bolted.

## Methodology

We report below on a series of empirical probes which bear upon the uses of F2F meetings in science. One data set consists of responses to questionnaires circulated roughly 6 months apart. The first was distributed by senior scientists in two domains of physics, using their regular mailing lists, in April 2020. In the UK, this coincided with the introduction of new legislation drastically limiting international travel and internal F2F contact, known as the period of ‘lockdown’. The two domains were gravitational wave detection physics (GW) and photonics (PH). Members of both groups were already aiming to cut down on science’s travel-related carbon footprint, GW having set up a committee to discuss it and PH having pioneered experimental remote conferences. In photonics, notably, there was great pre-existing interest in reducing conference travel (e.g. Achten et al., 2013; Reshef et al., 2020; Sanz-Cobena et al., 2020), culminating in the Photonics Online Meetup in January of 2020, in which 1100 researchers met in local F2F groups connected to other groups remotely.^12^ As one PH researcher put it in a personal letter to the authors, ‘we can finally start including all the bright minds from around the world which have, thus far, not been allowed to join due to either financial or visa hurdles’.

In the first questionnaire, there were four sections, the first three of which were identical for both domains. Section 1 covered basic questions about individuals’ professional status and identification for a comparative follow-up questionnaire. Section 2 covered respondents’ current view of the switch to remote communication and was mainly quantitative. Section 3 was the longest, based on open-ended questions on why scientists attend conferences, reasons for travel, frequency of attendance, previous in-person and online attendance, examples of conference experiences, and the making of trusted friends. Section 4 was again short and for PH was only addressed to attendees of the Photonics Online Meetup, asking about their experience and how they attended. Section 4 for GW asked questions on how two newly acquainted communities of scientists integrated, specifically those who joined the GW domain since the 2016 discovery of gravity waves or who were long-standing members of the gravitational wave project (from 2005 or earlier). The second questionnaire was circulated via email in November and December 2020, after 6 months of lockdown. It showed individuals their answers from 6 months earlier and asked for changes of opinion based on intervening experience.

Though the questionnaire method is generally associated with quantitative research, the empirical work reported here is essentially qualitative. This is why we talk of *probes*. Probes are appropriate when the characteristics of the domains being investigated are so uniform that the statistical representativeness of a sample is not an issue. The two domains each comprise a thousand or so scientists who are used to meeting regularly in the region of five times a year and can therefore be thought of as sharing a common social world like a small tribe. Many of the quotations are followed by information on the status of the informant and this also indicates that understanding of the social nature of the group is consistent across different roles and status levels within the domain, though younger members will be less advantageously immersed in it. An implicit assumption of uniformity applies across participatory methods in the social sciences or wherever the term *native informant* applies.^13^ The uniformity is in the experience, not necessarily in the opinion it gives rise to: for example, the common negative experience of apprentices that will be discussed later could still leave some deciding never to fly again even though it might damage their careers, but here we are not engaged in trawling opinions but revealing a common world. However, given the pandemic and the constraints of time and resources, the questionnaire *form* was appropriate for the probes conducted in April and November 2020, and again the responses should be seen as the remarks of an unusually large number of native informants: there were 41 and 35 for GW and PH, respectively, in April, with 35 and 21 follow-ups in November. We will also quote some remarks gathered by Collins during interviews at a GW meeting in Budapest in 2015, asking why scientists came to conferences rather than relying on the internet.^14^

These results are set in the context of earlier participatory studies including Collins’s 45 year-long immersive study of the detection of gravitational waves (GW), during which he attended scores of conferences and demonstrably acquired the language of the domain through immersion at such events, and H. Collins’s (1974, 2001) analysis of the transfer of tacit knowledge both for building TEA-lasers, and for measuring the quality factor (‘Q’) of sapphire.

Fortunately, in the case reported here the cultural gap between analyst and the experienced academic readers of this paper is not large and for these there should be none of the problems associated with understanding remote societies with incommensurable world views. This paper is about changes to conference-going practices and many readers of this paper are themselves conference-goers who have experienced the recent transformations: these readers will, for example, have learned of the surprising advantages of Zoom-like platforms and the advantages in respect of distant colleagues as well as the problems caused by the lockdown, and they share the culture of academics who are engaged in global communications that cut across regular cultural differences. So, these experienced academic readers can gauge the verisimilitude of the physicists’ remarks and the overall argument’s epistemological warrant. At the same time, it should be born in mind that natural scientists are not sociologists and remarks that bear on that part of the argument that deals with the need for common scientific ‘socialization’ across national cultures, and how this is the long-term foundation of science as we know it, are unlikely to be spontaneously volunteered: few natural scientists are continually thinking about the nature of scientific socialisation in the way that sociologists of scientific knowledge think about it as part of their professional duty.^15^

## Results

We can draw a few quantitative conclusions from the questionnaire responses: in both domains, scientists travelled to roughly five conferences per year and reported roughly 2–3 advantageous encounters (leading to new collaborations, etc.) every 10 meetings – or about 1.25 per year. We know, however, that there are large variations in meeting attendance. For example, Nobel Laureate Barry Barish told Collins in 2020:

I looked at my calendar for last year, and I took 36 trips last year having 51 different stops. The **main** reason for the trips – about 1/2 were to give some sort of talk, 1/4 committees, etc., and 1/4 direct research trips. It is disturbing to me that only 1/4 had to do with doing research as the main reason. Being isolated for ~10 weeks, I have given 4 talks by Zoom, participated passively in 20 LIGO meetings, and worked on direct research 26 meetings. Somehow, more research fraction, but it is misleading, because those are more passive meetings than [they are] research trips.^16^

There was also some indication that PH scientists felt more strongly about the need to reduce carbon emissions but, given the small numbers and general approach taken here, this difference invites further research.

### The uses of face-to-face communication

Section 3 of the April 2020 questionnaire, comprising mainly open-ended questions asking about the benefits of F2F, proved the most useful and is our focus in this section, alongside some responses from fieldwork conducted in 2015.

Face-to-face meetings have immediately evident uses as well as major consequences for the nature of science and of society. Table 1 attempts to pull together all the roles and advantages of F2F meetings in science. This table represents the analysts’ view bolstered by the literature, by reflecting on our own conferences and attendance at GW conferences (e.g. reported in H. Collins, Evans, et al., 2022) and drawing on the Budapest and more recent questioning of respondents. There are six broad groups of uses, starting with the most abstract, which are most likely to be stressed by sociologists of knowledge and only rarely mentioned by scientists, and proceeding to more concrete advantages, more frequently found in the remarks of natural scientists. We hope Table 1 will be a useful starting point for discussions and disagreements about the use of F2F meetings and for debate about what exactly can be replaced by remote interaction. We will explain each item in turn below the table and illustrate with quotations from respondents as far as we can (there will be some overlap in the substance of the quotations and the categories).

**Table 1.** Uses of face-to-face communication in science.

I Forming a sub-society
1. Energize/build trust, by being willing to spend time and resources
2. Transfer tacit practical skills – an element of contributory expertise
3. Transfer the domain language – interactional expertise
4. Be immersed in the linguistic corpus – the sounds and silences
5. Celebrate discoveries and legitimate their founding assumptions
II Trusting individuals
6. Personal acquaintance leads to trust
7. Sharing food and drink (commensality) leads to trust
8. Trust leads to commitment and the risk of time and resources
III Using presence to create and modify meaning
9. Body language conveys nuances of meaning
10. Co-presence enables safe and cognitively productive disagreement
11. Settle disagreements quickly and efficiently
IV Using presence to promote efficiency
12. Arrange multiple small groups
13. Meet many individuals
14. Likelihood of serendipitous meetings
V Building careers and collaborations
15. Flatten hierarchy by meeting ‘big names’
16. Arrange studentship and post-docs – build new networks
17. Arrange new collaborations, often serendipitously
VI Maintaining the businesses of science
18. Gather funds to support scientific societies
19. Facilitate collaboration with industry

#### Category I

The first category is socialization into a scientific sub-society. If a novel domain is being formed, the very act of traveling to a single location for the purpose of discussion is energising – people are showing their willingness to countenance the new collective idea by spending their time, energy and, perhaps, money. In the same way, a newcomer to an established domain can exhibit commitment and therefore earn trust – Collins noted this from the way he gained trust in the GW domain. The process of socialization into an experimental scientific domain to the level of *contributory expertise* will involve the transfer of the tacit knowledge needed to execute experiments, which can only happen F2F.^17^ Fluency in the *domain language* will also have to be acquired – this is *interactional expertise* – and acquiring it is a F2F process just like acquiring fluency in a natural language. Acquiring interactional expertise in a science (or any society!) involves the tacit transfer of background assumptions. Part of this tacit understanding is acquired from embedding in the corpus of language – that is to say, in the distribution of sounds and silences that frame thinking about persons and physical objects in the field. This is illustrated in the changing reputation of GW pioneer Joseph Weber (his name became less and less mentioned) and the changing status of black holes from theoretical entities to supposed ‘discoveries’ long before they had been seen.^18^ There is also a celebratory role for large conferences where individuals are awarded prizes or prestigious recognition (such as publicized plenary sessions to announce new findings) and, through audience response, establish acceptance in the wider community – the community reveals its willingness through applause and by withholding criticism it establishes the necessary new background assumptions (which may well not be accepted by the ‘fringe’).^19^

Though the cultural role of scientific meetings was not at the forefront of respondents’ reflections, there are instances in both the 2015 Budapest interviews and the April 2020 questionnaire responses:^20^

Part of being an expert is understanding whether what’s being said is legitimate, what result people believe and what they don’t believe and what’s out of fashion, and you can’t get that unless you are around other people and you’re seeing their reactions. You’re sitting at a table at lunch and you bring out some paper or some theory and you can see how everyone reacts. And if you’re writing letters to them asking them [that won’t happen] … (GW [Budapest])^21^

Attending one of the “crackpot” sessions at APS [American Physical Society] reassured me that even though I may have crazy ideas, I was still operating within community norms and mores. I could see the boundaries of physics more clearly — and accept them. (Postdoc, GW; 11–15 years’ experience)

The following comments from April 2020 also bear on socialization in an indirect way through the senses of community, culture and identity that reveal the processes of enculturation:

Understanding colleagues as human beings (related to “building trusting relationships”); feeling part of a community. Re-authenticating oneself as a scientist by integrating shared values, harmonizing and reconciling with the personal growth (e.g. thesis/dissertation, long hours at work) and sacrifices (e.g. distance from family, two-body/dual-career struggles) needed to participate in the scientific enterprise. (Postdoc, GW; 11–15 years’ experience)

I miss the cultural exchange – of going for a walk together, or to visit a cathedral, or to eat together. For many meetings I have been to in the past these are the aspects that have been most enjoyable. They have given me a shared sense of cultural and intellectual identity. If I had not had opportunities to do that over a period of years, albeit much less than I did, I wonder if I would have such a strong sense of identity now? (Professor of Photonics, PH; 31–40 years’ experience)

I think the resolution is less of a one time aha moment, and more of a slow osmosis of absorbing the LIGO culture. [LIGO = Laser-Interferometer Gravitational-Wave Observatory.] This osmosis happens faster at face to face meetings, so they are useful, but it happens in online meetings, telecons, and by email as well. And even at its fastest, it takes years. (Associate Professor, GW; 21–30 years’ experience)

#### Category II

The next category concerns the process of learning to trust specific group members and their claims. The transfer of the ability to measure the ‘Q’ of sapphire is an icon of the development of trust in a named scientist and there is also discussion with examples in the account of the 5 months of confirmation of the first direct detection of a gravitational wave. This feature came up regularly in the data, including from 2015:

When the collaborators are in different places you can have tensions and misunderstandings can build up. And often you get really frustrated with someone and I think they are doing things wrong and they’re misunderstanding things and maybe we’re just not going to continue working together because this is not going well. And then you see them in person and you have a beer and you chat about things and then it’s all fine. If you don’t have that every so often, things can get very complicated – for no good reason – just misunderstanding – paranoia … Maybe they’re trying to get to a result before you, or they’ve got some hidden agenda. To some extent we’re all trying to do this, we’re all trying to get ahead, but the fact that you are working together and you respect each other and you want to continue working together – that doesn’t come across in a telecon[ference] or certainly not in emails … I think people who don’t go to meetings and stay away – I think they get wound up in their own world. (GW [Budapest])

I was presenting measurements of charged defects in a particular semiconductor. This was an important topic since this material was on its way to commercialization. I, and the presenter just before me, presented contradictory results. We spent the next 2 days discussing and debating. Although we didn't come to a final conclusion that week, this encounter spurred us both to verify our experiments and within a couple of months, there was consensus. Without face to face discussion, which allowed each of us to gauge the legitimacy of the other's position, this disagreement would've bounced around the literature for an extended period to[sic] time, possibly years. (Materials Research Engineer, PH; 6–10 years’ experience)

Got face time with important and famous scientists at meals; had informal conversations that allowed me to assess the research directions others were proposing. (Assistant Professor, GW; 25–34 years’ experience)

One comment from April 2020 stood out as reinforcing the importance of F2F interaction specifically for developing trust:

I’ve met people through online tools, but I can't say they are trusted friends, as I haven't met them face to face. (Post-doctoral researcher, PH; 5 years’ experience)

We note that sharing food and drink (often referred to by analysts as *commensality*) was frequently mentioned, and in many more excerpts than we could list here, it also being an iconic element in the transfer of the Q of sapphire.

#### Category III

This category identified the extra meaning transmitted and modified through body-language, and the trust that physical presence engenders. Respondents argued that F2F enabled safe, vocal, and cognitively productive disagreement:

I think sometimes it’s useful to have this role-play where you say something and I deliberately try to find arguments against it, though my instinctive reaction might be to agree with you but doesn’t help, so I try to be critical and I say ‘no – why isn’t this and this?’ Some of the very best discussions I’ve had – some of the best interactions and experiences in science are in a small group of people when you can say ‘no that’s wrong’ and you can argue about it, and there is a sense of trust the person doesn’t think you’re an idiot: they know you’re smart and you’re confident and you’re asking a real question, and they’re honestly trying to explain it to you and you’re honestly trying to understand it, and from that ideas flow and one of the most interesting results, the XXXX thing, came from a week of – there were a few of us – and at the beginning of the week someone says, ‘I think the thing looks like this’, and then someone says, ‘I think it looks like this, no it’s not like that’, and back and forth and arguing, and within a week we’d found something new that we truly didn’t expect at the beginning. (GW [Budapest])

This is a sensitive example. While at a conference, I encountered several researchers who confided that they were extremely skeptical about certain published results. Face to face interactions allowed us to air these worries candidly, exchange possible explanations, and discuss methods to verify the results. It is much less likely that one would state these concerns as openly in an email, which would leave a formal record, or discuss them in an online forum that would include many unknown participants. (Materials Research Engineer, PH; 6–10 years’ experience)

Had a frank but productive disagreement with a much more senior scientist, which would have been much more daunting and less easily resolved if it hadn't been face-to-face. (PhD student, GW; with 2 years’ experience)

There are always collaborators who get easily miffed at the slightest hint of offense (even if none was intended). Digital and remote communication are terrible at dehumanizing folks to the point where you're barely even communicating with each other. There's really something about sitting next to someone, letting them have it out with you, and getting the chance to explain oneself – with body language included – that really helps smooth things over. (Control systems engineer, GW; 16–20 years’ experience)

There is something important about in-person meetings, where body language can be read and expressed, where you can go on social outings, that is hard to replicate remotely. Even questions asked following remote talks are fewer in number, shallower in content, and harder to engage with than in-person questions. (Assistant Professor, GW; 11–15 years’ experience)

No. I need a *whiteboard.* I need to *point* at things. I need to read the body language of my audience. I need them to feel comfortable interrupting me. All of these things cannot happen (or at least are very difficult to facilitate) without the face-to-face. (Control Systems Engineer, GW; 16–20 years’ experience)

#### Category IV

Respondents also noted the sheer efficiency of gathering in one place – the efficiency of personal interchange, the ability to have many personal interchanges with many people over a compressed time scale, and the possibility of serendipitous meetings:

Here you can just go round a bunch of people and in ten minutes the whole thing [a new collaboration] is arranged and you can tell immediately whether the people are enthusiastic, how interested they are, how much commitment you are going to get from them just from their reaction, which you would have no idea about over email. In email [you get]: ‘that’s great! I’d love to do that’ [but it does not mean anything.] (GW [Budapest])

LSC meeting in Germany. I was presenting some of my thesis work to the continuous wave search group at a F2F breakout, which I was essentially submitting after the LSC meeting. It was very useful to get in person feedback, as well as simply walk to a blackboard and answer some questions about it. What might have taken a few weeks in e-mail chains back and forth could be gotten over an hour in a conference room. (Research Physicist, GW; 16–20 years’ experience)

At a LISA cosmology group meeting, had a session specifically set aside to work on an ongoing paper, which had been dragging on for months with little progress, due to a large number of authors not communicating efficiently remotely. We had a heated discussion which resolved a lot of issues and brought the paper a lot closer to completion. (PhD student, GW; 2 years’ experience)

#### Category V

The fifth category includes flattening hierarchy and arranging studentships, post-docs and other collaborations. ‘Flattening hierarchy’ refers to making distant figureheads real and in arranging studentships and other kinds of apprenticeship-based collaborative work. This is the kind of thing that is very much to the forefront of the reflections of scientists, especially young ones:

At the Einstein Toolkit Workshop 2019, I had the opportunity to meet in person those whose research I had studied and whom I regarded as influential/famous in the GW domain, which had the effect of flattening hierarchy in allowing me to discuss with them. (PhD student, GW; less than 1 year experience)

I met a very big boss of my field, who turned out to be a nice guy. I am writing a EU project with him and my best master’s student ever is doing his PhD with him. (Associate Professor, PH; 16–20 years’ experience)

I met a peer colleague who left the lab I was working in before I joined it. I always heard of him, I could never talk to him. Now he is one of my best collaborators. (Associate professor, PH; 16–20 years’ experience)

Gravitational Waves and Cosmology workshop/school in Brazil. It was a 2 week workshop aimed at creating interest and basic introduction to the GW field for undergraduate students, graduate students, and post-docs in South America. I don't think such a conference would have been nearly as effective for the students in a remote format, given the discussions we had outside of the nominal talks/lectures. Similarly, questions asked by some students were about the nature of the collaborations, competitiveness, working at the sites, and so forth, which might have been even more awkward online rather than in person over dinner, for example. (Research Physicist, GW; 16–20 years’ experience)

Photonic conference, watched a presentation before mine, on material science, and got intrigued by the material that was presented. after had a chat with the speaker, got a sample, started a collaboration. now 5 years later it is one of my main research lines, it was the material I needed to develop the network lasers I wanted to explore. Pure serendipity. (Reader in physics, PH; 11–15 years’ experience)

Invited at a conference, and [having]given my talk I started wandering into different rooms. Went to a talk in a different field, and after started discussing with the presenter, now we have funding together and a great collaboration (Reader in Physics, PH; 11–15 years’ experience)

[I met XXXX] (USA). I had never met him before - we are now working towards a collaborative project. We got to know each other in the queue waiting for a conference dinner. (Professor, PH; 31–40 years’ experience)

At conference by the sea in San Sebastian, I made two very good friends (in the breaks, not in the sessions!). With them I co-organized a series of meetings and I wrote 20++ papers and I have a EU project approved. Very valuable. (Associate Professor, PH; 16–20 years’ experience)

#### Category VI

The final category concerns the business of science and is only indirectly about scientific knowledge. Nevertheless, it must be understood if we are to consider the way that science might change if F2F conferences and meetings were to be abandoned. Large conferences raise money through registration fees to support scientific societies and, more notably in some sciences than others, foster interaction between science and industry in the trade fairs set aside for manufacturers displays. In physics there are annual meetings of this kind (such the American Physical Society) but these concerns did not feature as prominently in respondents’ comments as they may in biological and medical sciences.^22^

### The 6-month follow up in November 2020

Contemporary sociologists’ conceptions of communication in science suggest that F2F meetings should be accorded a very high value. The active campaign for a shut-down of international conferences by some scientists suggests that in these circles the cultural aspects of science are not a salient feature or the role of F2F communication in building culture is not understood in the model of acquiring fluency in a language. Though it would not change the sociological analysis of the first part of the paper, we still wanted to know how the actual experience of the pandemic had affected physicists’ appreciation of meetings. Would it turn out, as some scientists hoped, that the forced experience of remote communication would encourage scientists to take advantage of its positive features and abandon F2F meetings altogether, or would the enhanced focus on what had been temporarily lost lead them to appreciate F2F interaction still more, even though the focus of their attention was unlikely to be on the cultural changes involved?

In late November 2020, we sent a short questionnaire, in the form of a simple email, to all those who had responded to the April survey receiving 56 responses all together. Questions 2 and 3 asked if, over the course of the lockdown, respondents had made any new lasting relationships or developed any new working relationships. The April questionnaire found that respondents reported around 1.25 advantageous relationships per year before 2020. In GW, we were looking at an aggregate of 17.5 extra years’ experience over the 6 months and we might have expected around 21–22 new relationship to have been formed had pre-pandemic circumstances been maintained, but there was a strikingly uniform ‘zero’ answer to Questions 2 and 3 from 34 out of the 35 GW scientists. Only one GW scientist reported forming new relationships since lockdown – three of them! Among the 21 PH scientists the expectation would be that around 13 new relationships would have formed during the 6 months, but 19 respondents reported none while the remaining two reported three new relationships between them. Six months is a short time in which to confirm that a new acquaintance or research collaboration has been formed and one or two respondents said that they were waiting for time to tell, so there might be some understatement going on here, but many respondents pointed out how hard they found it to form new working relationships or friendships through online interaction alone.

The most informative results to the November exercise were the responses to Question 1 (Have you learned anything from the experience of lockdown that has caused you to change your mind about the relative value of face to face and remote meetings?) and Question 4 (Are there any other specific gains or losses that you have noticed since face to face meetings ceased that have not been mentioned so far?). The entire 8,600-word set of 56 replies may be inspected and independently analysed at https://osf.io/27wnh but it is too long to publish within a research paper. Here we will try to exemplify and describe it. We will finish with an impressionistic summary constructed by giving rough numerical scores to the responses. Table 2 shows examples of quotations where they match the categories of Table 1 in interesting ways.

**Table 2.** Categories from Table 1 illustrated by quotations from the 6-month follow-up.

I	Forming a sub-society
	**Energize/build trust, by being willing to spend time and money: GW19:** I think because online meetings are easier to organise than physical meetings (just invite people and set up a zoom link), there seems to be much less thought and effort put into them, meaning that the whole experience ends up being less useful
II	Trust between individuals
	**Sharing food and drink (commensality) leads to trust: PH3:** Face to face will unlikely be phased out simply because people are more likely to network over coffee, a meal, glass of wine (beer) etc. or simply by the random act of sharing a space near someone you have not met before
III	The use of presence to create and modify meaning
	**Body language conveys nuances of meaning: GW25:** Judging the impact of a delivered talk at a remote-only meeting is challenging, as there is no way of knowing if the audience actually paid much attention. **GW26:** Without seeing their face (nobody likes to show video) and their gestures it is difficult to form an intuition about their true intent
	**Settle disagreements quickly and efficiently: GW38** There are too often specific questions/problems coming up that require a side discussion between subgroups, there are also political/social issues that are easier to discuss if you can see the body language of your colleagues
IV	The presence of the body to promote efficiency
	**Arrange multiple small groups: GW27:** We use to joke in the hallways, as we left in-person meetings with large groups that were intended to be coordination meetings, that OK, so now you want to really plan what we’re going to do? And then solidify our plans in the following 5–10 minutes conversations. That can’t happen anymore!
	**Likelihood of serendipitous meetings: PH18** But I need the spontaneous discussion about a topic with someone else. I think the spontaneity can only happen in person when you let yourself time enough to interact with someone. I think this is particularly important in Science and I feel that I am less creative in this situation than I was just a year ago
V	Building careers and collaborations
	**Arrange new collaborations, often serendipitously: PH4:** besides establishing the “obvious” collaborations, we are not establishing new ones from the serendipity of conference meetings. Our team meetings are way less efficient, and there are often miscommunication issues
VI	Maintain the businesses of science
	**Facilitate collaboration with industry: GW14:** My current research depends crucially on a relationship with a vendor. The person I collaborate with at the vendor has been accessible by zoom and that has not been a problem. But the small company was bought out by a larger company, and I do have concerns about the larger companies commitment to working with LIGO. A trip to the larger company to give a talk, meet some people, just make us seem more real to them would make sense. But of course it is not possible. Hopefully it won’t be a problem. But if this work flounders because of the relationship with the vendor, I will probably always wonder about the role of COVID on that

Bold identifiers (‘GW19’, ‘PH3’, etc.) indicate source quotation in full data set at https://osf.io/27wnh.

We will also show that the results indirectly support the claim, made early in the paper, that the profound effects of a shift to R2R may not be felt for a generation. The responses reveal an unprompted and unexpected emphasis on the negative effects of the suspension of F2F interaction on new entrants to the domains. Around a third of the responses to Questions 1 and 4 combined stress the damage specifically to new entrants.

### Responses and scoring

We gave an impressionistic numerical score (ranging from −2 to 2) to each of the 56 responses in respect of their experience of R2R and F2F respectively. A score of −2 represents a strongly negative report, 2 represents a strongly positive report, with 1 and −1 representing qualified positive or negative reports. Absence of a comment, an indecipherable response, or an even balance of positive and negative scored 0. We considered only the impact of the shift from F2F to R2R on the way scientific knowledge is made, so our scores do not consider comments regarding carbon footprint and the like. Likewise, we ignored claims that future technological changes would resolve the remaining differences between R2R and F2F. Reports of F2F and R2R were scored independently, so a respondent could score −2 for both reports of R2R and F2F, or 2 for both, or any other combination. The Appendix gives examples of how we scored the responses.

**Figure 2.**
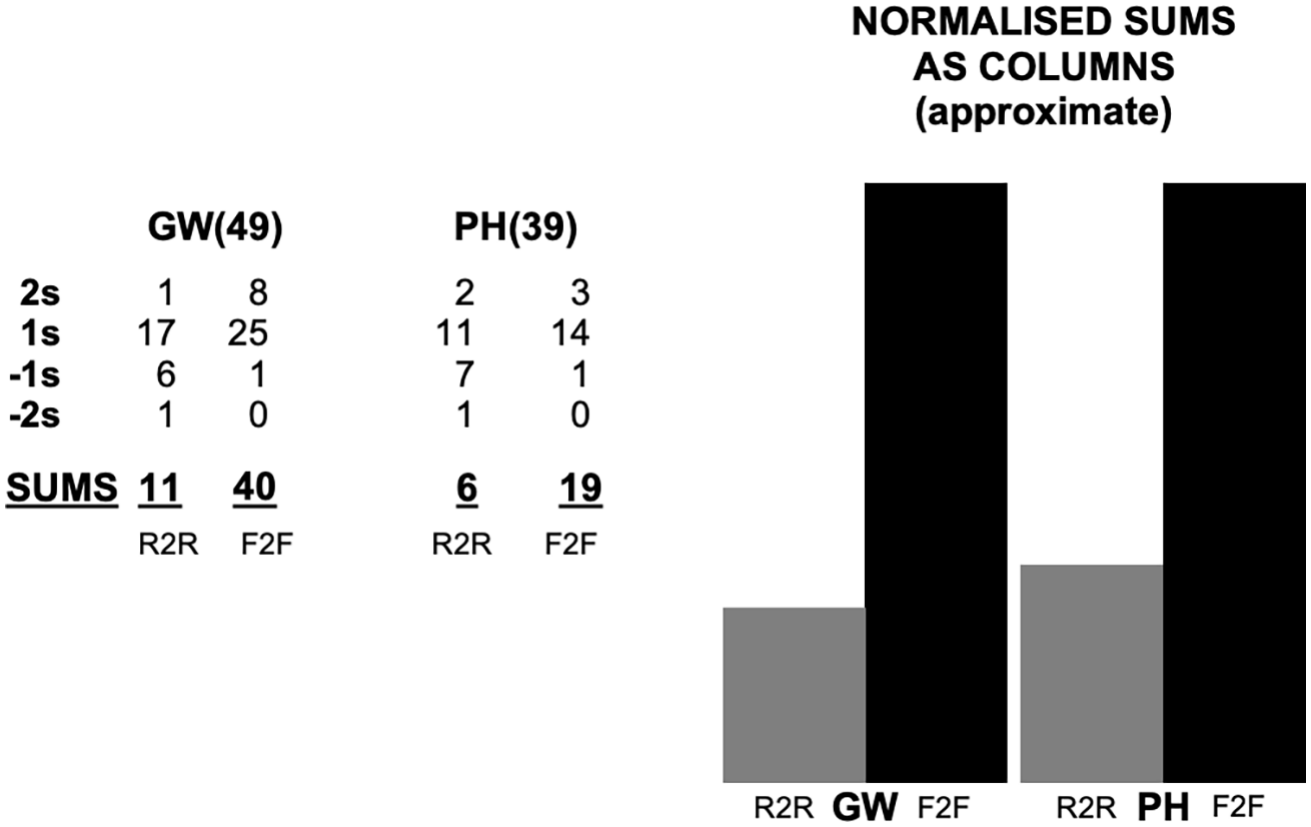
Respondents’ reports of experiences of lockdown in summary.

Figure 2 shows the way we distributed scores (ignoring 0s), across the whole November data set, and also illustrates, in normalized column form, the way responses compared. The two left-hand columns are for GW, the leftmost being for R2R and the other being for F2F. The two right-hand columns are for PH.

Of course, discursive remarks can be scored in different ways, and we tried an alternative in which we assigned negative and positive impressions to *each phrase within a reply* and took the balance. This had almost no impact on F2F but reduced the score of R2R in GW well into negative territory and reduced it to roughly zero in PH, reinforcing the overall interpretation offered here: F2F is missed almost irrespective of starting opinion and R2R has been met with mixed feelings even though there have been positive surprises.

Most respondents reported that they had been surprized at how effective R2R had been, accounting for the *overall positive initial impression* even in the left columns. This seems to reflect a widespread view that the technology of Zoom and similar platforms is far better than the Skype or the telecons that were widespread before the pandemic and that under lockdown conditions, Zoom-like meetings are far better than no meetings; they enable work to continue, even with some advantages where distant colleagues are concerned, whereas without such platforms it would have been much harder.

PH respondents were slightly less enthusiastic about F2F than GW respondents, and it is tempting to relate this to their relative position on the X-axis of Figure 1, but the numbers are too small to do much more than look forward to future work to confirm any such difference. Answers to Question 2 in the follow-up could also be revealing of some cultural differences between GW and PH in ways that correspond with the categories of our theory and may warrant further investigation.

### Impacts on new entrants to a field

As noted above, many responses to the second questionnaire pointed to the severe impacts of socialization restrictions on scientists in their field at an earlier career stage. The first two of the following quotations are from established scientists who know how the world works in their scientific domain and can therefore speak authoritatively on the impact on new entrants. The three latter quotations reflect these views in the words of more junior scientists experiencing what they feel to be problems early in their careers. Most of the reservations are expressed in terms of career-building but there is also an intimation of the need for peer-groups and networking:

My new PhD student is finding this really difficult. At a meeting I could introduce them, and their face/name would be recognised the next time around. However, a lot of meetings have stopped even showing the participants because the list is too large. (‘GW29’, Senior Lecturer; 11–15 years’ experience)

My student has lost opportunities to network in preparation for a job search. She is the kind of person whose abilities are very apparent in person, but at the moment less obvious on paper. I feel that her job search has been hindered by not being able to travel and meet people or give talks. (‘GW38’, Assistant Professor; 11–15 years’ experience)

It is not easy for early career scientists to have informal talks with more senior colleagues from other institutes at online meetings. These talks are quite useful as they may lead to collaborations and/or job opportunities. (‘GW28’, Postdoc; 5 years’ experience)

I think that for people further along in their career, remote meetings are fine, because they do a good job of relaying information. However, for younger professionals who still really need/want to build their networks, face to face meetings are so much better for that. It’s a lot harder to ask someone to have lunch or coffee over zoom than it would be at a meeting in person. (‘GW35’, Postgraduate Researcher; 5 years’ experience)

I have had to rely on my supervisor's network of contacts since I have been unable to build my own. (‘PH11’, Postdoctoral Research Fellow; 6–10 years’ experience)

The Z-axis – the time dimension – of Figure 1 is collapsed by new entrants because they have no existing trust-networks to rely on and they cannot build them in the absence of F2F. This means that we are effectively looking at the future through the experiences of new entrants. As one senior respondent put it:

I suspect that our success depends critically on having established in-person relationships in the past with the vast majority of the persons with whom we are now interacting at a distance. I think new members (tend to be early-career, diverse, more fragile, more easily discouraged and more in need of short-term positive reinforcement) are at a very strong disadvantage, and there will be a continual degradation in the quality and quantity of science as people move around. We need a recharge of in-person meetings; it remains open how frequent, broad, and sustained that ‘recharge’ must be to bring in and support new participants. (‘GW15’, Senior Research Scientist; more than 40 years’ experience)

## Conclusion

We have looked at the impact of the Covid-induced restrictions on socialization on two domains of physics. We have found that, overall, the experience has caused these scientists to qualify their enthusiasm for R2R conferences and meetings and come to appreciate the advantages of F2F more clearly. Some scientists who were approached in our November follow-up, especially from the photonics group, were at the outset of the study in April 2020, extremely critical of the existing situation:

I sincerely believe that the lockdown has enabled the online conference concept to be tested, since the appeal for reducing our contribution to climate change did not sway the general community. I think the silver lining of this pandemic situation is that more conferences will switch temporarily to an online format which I find brilliant. On the one hand, the amount of time saved by not having to travel, apply for visas, is outstanding. On the other hand, the cost of conference attendance has drastically dropped, opening up for more people from different communities the opportunity to expose their ideas! I call this win-win, that means more time and money can be spent in what really matters: research. (Oberassistent, PH; 6–10 years’ experience)

But in November 2020 even this scientist noted:

I still think that you need at least need some element of face-to-face meetings to introduce this notion of randomly encountering/interacting with someone you otherwise would not.

Our theory, represented in Figure 1, suggests there will be little noticeable difference to science in the early stages of any sudden transformation (such as pandemic-induced travel and gathering restrictions), as the effects of socialization and trust-building will not degrade for some time. To understand the full social impact of such a change, it is necessary to imagine a generation of scientists who have never experienced F2F meetings outside their own institutional locations. That said, the potential impact on the initial socialization and community networking of apprentices is already becoming evident to our respondents, as might be expected for newcomers to the field who do not have a foundation of dense trusting relationships (those located at the foot of the Y axis of Figure 1). Scientists who responded to the November exercise – both juniors in search of PhD or post-doc positions and their potential supervisors and employers – made the point many times that difficulties in this regard had become forcefully apparent to them.

A satisfactory solution could be what one professor of photonics referred to as a ‘mixed economy’:

I envisage a ‘mixed economy’ as we move ahead - and in many ways that’s a positive thing … We see more clearly now what [are] the real values of face to face, and should be better placed to ensure we use such opportunities more fully, and to best advantage for all. (Professor, PH; 31–40 years’ experience)

**Figure 3.**
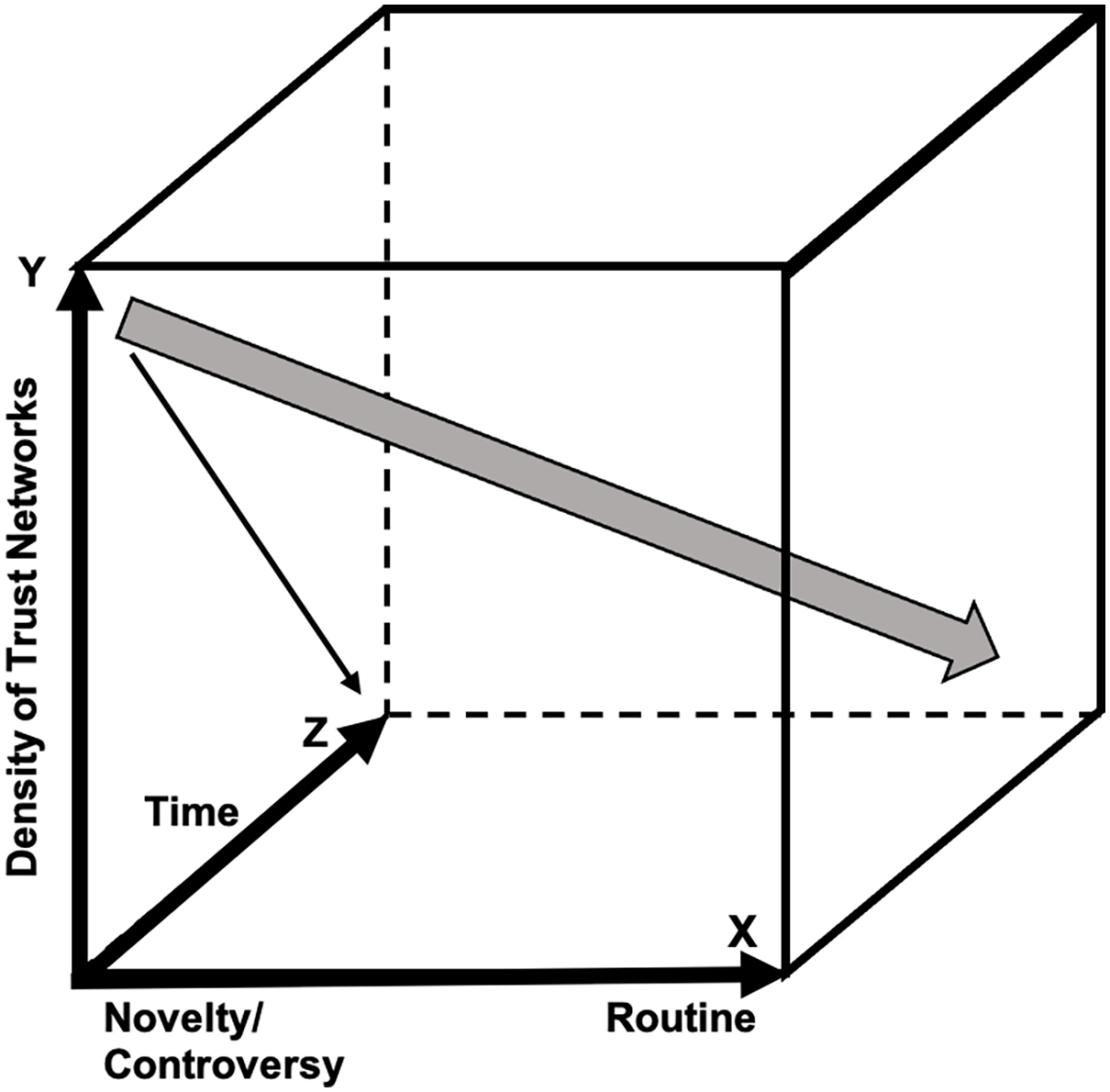
Possible consequences of a reduction of trusting relations with the abolition of F2F.

A wholesale shift to R2R raises more depressing and more portentous possibilities. Science would have to rely less on interpersonal trust. Some possible consequences are represented in Figure 3.

The thin arrow sloping down the left-hand face of Figure 3 represents the diminution of trust as F2F disappears over time. One response could be that science will become less adventurous and more routine in the search for reliability and, as indicated by the thick arrow, finish up in the bottom-back right-hand corner: science will stagnate. Or the opposite may happen: science will become radically more adventurous as truth via argued consensus among trusted colleagues becomes replaced by social media-type interaction involving unknown but popular contributors and science becomes dominated by adventurous speculation supported by media celebrities. A consequence might be that science will become less universal as interpersonal guarantees of trust are replaced by proxies such as social class or its modern equivalent, prestige of institutional affiliation. Ironically this would mean that what appears to be a potential for increased egalitarianism through the abolition of meetings that some find difficult to attend, will play out in the form of still less egalitarianism in respect of remote locations. If the proxies for interpersonal trust relations come to include celebrities of various kinds accumulating ‘likes’, as represented by the bottom-back left-hand corner of the space in Figure 3, then science will become harder and harder to distinguish from popular cultural institutions such as social media.^23^ Some of our respondents did indicate that in adapting to the loss of F2F they were using social media *more*:

I think I need to be way more active on social media (e.g. twitter / linkedin) now that I have fewer “chance meetings” at conferences (PH Postdoctoral Fellow; 6–10 years’ experience)

It will be very hard to make new friends. Probably will build on past acquaintances and collaborators. Follow more people on LinkedIn and Twitter and start participating in their online posts and tweets. (PH Research Associate; 6–10 years’ experience)

Existing research indicates that fringe beliefs are much more readily supported by social media and other internet-based interchanges than the dense F2F groups typical of science. If science’s truths become local rather than universal, no longer cross-cutting national and other social boundaries, the existing elite institutions will be strengthened, unrelieved by even the possibility of talent from less well-known institutions crossing institutional boundaries. In these circumstances, science would no longer be science as we know it: it would be unable to take a leadership role as an exemplar for truth-led decision-making in democracy, a sad prospect for pluralist democracy as a whole, as science’s role as a check and balance will lose credibility in the way populist leaders would prefer.

The current conference circuit almost certainly ought to be modified to take account of the criticisms at the heart of the anti-conference movement, but we argue here that abolition is not the answer. A possible solution is the ‘mixed economy’ referred to above to address the different concerns such as increasing accessibility and tackling inegalitarianism in some of the ways a number of our respondents had raised. For those who currently feel the impact of climate change imposes an immediate moral obligation, a solution could be to ration long-haul travel to those meetings where a significant amount of time is set aside for personal interaction; taking such a moral obligation seriously would imply that busy celebrities who can appear only to present a paper at a conference and depart immediately should restrict their conference role to remote appearances. To introduce such changes to entire communities of scientists, efficiently and without unexpected and unwanted consequences depends on a more complete understanding of how the sciences work and how this differs between adventurous and routine sciences. In terms of resolving inegalitarianism, we need to think carefully about what opportunities would be lost by abolishing all personal contacts outside of home institutions, which are themselves sometimes the source of elite-driven inegalitarianism.

## Supplemental Material

sj-docx-1-sss-10.1177_03063127221138521 – Supplemental material for Scientific conferences, socialization, and the Covid-19 pandemic: A conceptual and empirical enquiry Click here for additional data file. Supplemental material, sj-docx-1-sss-10.1177_03063127221138521 for Scientific conferences, socialization, and the Covid-19 pandemic: A conceptual and empirical enquiry by Harry Collins, Willow Leonard-Clarke and Will Mason-Wilkes in Social Studies of Science

## Notes

1. Virtual/remote conferencing includes a range of practices and activities (Anderson & Anderson, 2010; Olechnicka et al., 2018). Attempts have been made to delineate some of the key characteristics and benefits/disbenefits of virtual/remote and face-to-face conferences (e.g. Hansen & Pedersen, 2018; Sá et al., 2019), and also to outline the emergence of hybrid F2F/R2R conferencing (e.g. Spilker et al., 2020).

2. Sarabipour et al. (2021) present what seems like an excellent analysis of the various kinds of inequity within the conference circuit, the sociology of knowledge is beyond that paper’s purview; its scientist-authors being medical or other kinds of life-scientists.

3. While virtual conferences have a reduced carbon footprint, it is not insignificant. Faber (2021) estimates that in 2002, a 6 hour, virtual conference with 207 attendees emitted over 1.3 metric tons of CO_2_-equivalent and this would increase linearly with attendees and duration.

4. Inequitable access along gender lines seems to be increased by a shift to R2R. For example, C. Collins (2020) shows that women are submitting less and men are submitting more conference papers with shifts to remote conferences during the pandemic, with women having increased caring responsibilities when working from home.

5. Another existing analysis of what seems to be a ‘science’ that fits the algorithmical model and may be close to hypernormal is the development of the SLICE rocket engine as described by Malhotra et al. (2001). It is said to have been very successfully and efficiently developed largely through remote communication (here we make no distinction between science and the frontiers of technology). Anecdotally, the strongest enthusiasm for the shutdown of the scientific conference circuit that we have encountered comes from scientists in the biomedical fields, though the suppliers of internet platforms are also likely to be enthusiasts for the power of their products as replacements for personal meetings.

6. This does not mean that certain scientists will not cheat or be more focussed on competitive success, merely that the main ‘formative aspiration’ of science must be to generate truth if the institution is to be recognisable as science rather than say, politics or business (H. Collins, 2018a; H. Collins & Evans, 2017b; H. Collins & Kusch, 1998).

7. Whether this testing is possible in practice is doubtful, but the aspirations are clear and important (compare them with the declared knowledge generation-related intentions of someone like Donald Trump or the Brexit campaign of Boris Johnson).

8. Members of cohesive and esoteric scientific domains frequently exhibit a variety of natural languages and dialects within them.

9. For the relationship between science and democracy, see H. Collins and Evans (2017b); for science as a check and balance for pluralist democracies see H. Collins et al. (2019). A longer manuscript outlining the relationship between kinds of democracy and kinds of communication, explaining why F2F is central to both science and democracy, is H. Collins, Evans, et al. (2022); this book also explores the way the internet can invade social spaces in the absence of trust relations developed through face-to-face interaction. The role of science in the maintenance of democracy has become far more evident under the Trump administration and the recent shift of political regime in the USA. These events are, of course, intertwined with the politics of disease prevention and vaccine hesitancy.

10. Historically, when personal contact between remote scientists was difficult and rare, trust was based on proxies such as social class (Shapin, 1994).

11. One damaging but unfalsifiable criticism that we have been disappointed to encounter is that the technology of remote meetings is only now being developed and will get better in the future. This, of course, is a standard response to criticism of new technologies.

12. For example, Achten et al. (2013) evaluates the carbon footprint of science, putting conference attendance at 35% of the footprint and argues that videoconferencing could reduce this up to 44%. See also Sanz-Cobena et al. (2020).

13. The explanation of the difference between probes and surveys and their roles in the social sciences is developed in H. Collins and Evans (2017a) entitled ‘Probes, surveys and the ontology of the social’ while the relationship between quantitative and qualitative is re-assessed in Chapter 9 of H. Collins’ (2019) book on sociological method. Nowadays, of course, it is hard to know what a reliable quantitative result is (Ioannidis, 2005). Here we will take the idea of a probe a little further, pointing out that the remarks of *unrepresentative* respondents have a place in the method too.

14. Collins’s 2015 study was aimed at explaining one of the reasons that artificially intelligent computers could not mimic the information transmission of human scientists, thus bringing sociology of scientific knowledge into the realm of AI. The results were first published as Chapter 8 in H. Collins (2018b).

15. Social studies of science is, nevertheless, lucky to have benefitted from the work of a number of natural scientists whose writings were based on sociological insights, an obvious, early, example being Fleck (1935/1979). One or two of the remarks recorded here are also of that exceptional type.

16. Barry Barish, 27 May 2020, personal communication to Collins, emphasis in original. We also learned from anecdotal remarks that in the photonics field too there are scientists who regularly present at around 50 meetings per year. In future studies, we hope to investigate the way frequency of conference-going is distributed in detail in different sciences.

17. As documented in H. Collins (1974) ‘TEA Set’ and H. Collins (2001) ‘Q of sapphire’ studies.

18. H. Collins (2004, pp. 451–452) for language and the reputation of Weber, and (pp. 452–453) for the status of black holes.

19. This ceremonial legitimating process was very evident at several conferences in the announcement of the discovery of gravitational waves (H. Collins, 2017).

20. Some responses have been lightly edited for English spelling or altered formatting.

21. More material from the Budapest interviews will be found in Chapter 8 of H. Collins (2018b).

22. This is based on Collins’s interview with Jim Woodgett triggered by his ‘Let’s break the cliquey conference circuit’ https://world.edu/lets-break-the-cliquey-conference-circuit/

23. These would be wholesale changes in the culture of mainstream science in terms of what Kuhn (1959) called ‘the essential tension’ between authority and adventure (revolutionary and normal science). That a shift towards novelty in the balance of the essential tension is a characteristic of fringe science is shown in Collins et al. (2017).
