# Supplementary material for: Scientific conferences, socialization, and the Covid-19 pandemic: A conceptual and empirical enquiry
Source: Soc Stud Sci. 2023 Jan 12;53(3):379–401. doi: 10.1177/03063127221138521 (PMC9841198; doi:10.1177/03063127221138521)
Supplement: sj-docx-1-sss-10.1177_03063127221138521 – Supplemental material for Scientific conferences, socialization, and the Covid-19 pandemic: A conceptual and empirical enquiry [file sj-docx-1-sss-10.1177_03063127221138521.docx]

## **Appendix**

The examples presented below illustrate the way we scored the responses in order to construct Figure 2. The examples also happen also to include sentiments **(bolded)** about the special effects on new entrants. Another theme (italicized) is spontaneity, serendipity, and creativity.

The following responses scored 1 (positive) in their sentiment towards both F2F and R2R communication:

Question 1: I have been surprised by the resilience of the community to COVID-19, virtual meetings have been surprisingly effective at maintaining existing collaborations**. I think lockdown disproportionality affects early career researchers as they cannot forge new relationships.**

Question 4: Yes, I started a new job in another country. Of course, I cannot travel to that country so I have developed new colleagues via Zoom all whilst working remotely. **Building these relationships has been slow, time-consuming and required both substantial effort and consciousness of the limitations of Zoom. Much more so than in-person meetings. I am involved with fewer projects than I expect I would have been, had I started in person.** (‘GW3,’ Postgraduate Researcher; 4 years’ experience)

Question 1: The lockdown time, and the last months, have allowed me to understand where face to face meetings are really required, and when remote meetings can actually be superior. I had many a priori ideas that turned out to be false. At the end, remote meetings turn out to be ideal for administrative tasks (project evaluations, department meetings, project meetings, etc.). They are excellent for short (i.e. 1-2 h) meetings with our collaborators, when the topic is well defined and slides and summaries can be prepared in advance. They are also surprisingly good for 1h scientific talks (colloquium style), allowing the younger members of the research teams to get in contact with the very top speakers worldwide. *Face-to-face meetings are required for weekly team meetings, for starting new collaborations with other groups (when it is not so easy to structure the meeting in advance), and for smaller seminars. Basically, they are needed when the direction in which the exchanges will go cannot be well planned in advance, and for the emergence of truly new ideas.*

Question 4: Gain: I waste much less time in some specific administrative duties (evaluation committees, administrative project meetings, etc.). We all became very used to the online format, so existing theory collaborations involving other countries are working way more smoothly than before (it is easier to fix online meetings). *Loss: besides establishing the "obvious" collaborations, we are not establishing new ones from the serendipity of conference meetings.* Our team meetings are way less efficient, and there are often miscommunication issues. Specific administrative duties become super hard to solve without face to face interactions. **We have completely lost the link to younger students (undergraduate, master), and this will become a longer-term issue for recruiting new team members.** (‘PH4,’ Professor and Group Leader; 16-20 years’ experience)

Other responses drew more distinction between the modalities, such as this, which scored -2 towards R2R and 2 towards F2F:

Question 1: I value face to face meetings much more now that they are gone. On the rare occasion, I am allowed to have them, I find I, as well as the other person, chat for much longer about subjects unrelated to work, though mainly related to COVID which is obviously a manifestation of social isolation. If anything, I have learned to hate remote meetings even more. Not just for their technical clumsiness, but the fundamental lack of human interaction that cannot be replicated on a screen. Emotion, body language and natural conversation flow are impossible. *Further to this, and vital for experimental research, is the fundamental lack of chance meetings/conversations. You simply do not "bump into" someone on zoom as you would in an office or corridor.* While I try to be open to new ideas, as an extrovert, and social person, I think I will never accept remote meetings as superior or even comparable to face to face meetings, the latter of which I desperately miss.

Question 4: Significant loss of mental health. Significant lack of progress due to no physical support from postdocs/other PhDs in the final year of my heavily experimental PhD thesis. Zoom/webcams are entirely insufficient/inflexible to provide meaningful demonstration of issues/discussions of experimental apparatus/samples for a good 60-70% of issues that I have. (‘GW4,’ PhD Student; 2 years’ experience)

While this one scored -1 towards R2R and 1 towards F2F:

Question 1: Since March 2020, I have participated in 4 online events of more than 50 people and not to a single "face to face" event. I have learned that the *online meetings rarely create lively discussions, those discussions that are necessary to build a scientific community*. Almost all of the discussions were driven by experienced researchers. **It now seems evident to me that the younger researchers would durably suffer with online meetings only as it is very difficult for them to meet these experienced researchers and also to interact with other researchers of the same generation. Informal discussions are so important**... More personally, I was happy to see talks of people that I wouldn't have seen otherwise (often because we live in different continents and I decided to minimize my journeys by plane), but I do not feel that I have learned much in the end. The proliferation of online meetings is clearly not a good thing in my view and I often felt that I had just lost my time. The classical format of conferences (keynote/invited/standard talks of fixed durations + questions, poster presentations in parallel, etc.) cannot be translated easily for online meetings. The chairperson should do its job for once (which is not just giving the name of the speaker, his/her affiliation and the talk title). As far as I have seen, online meetings cannot replace face-to-face meetings. They offer new possibilities (listening to someone living on the other side of the world) but are not sufficient by themselves.

Question 4: The main positive effect is a reduced emission of greenhouse gases due to less flights, when these are not compensated by the emissions due to heavy data usage. Face-to-face national meetings, with all participants coming by train, may perhaps emit less (??) than the same meetings if hold online, depending on the number of participants, event duration, etc. (‘PH8,’ CNRS Researcher; 11-15 years’ experience)
